# How Leader Narcissism Links to Team Voice Behavior: The Mediating Mechanisms of Leader Voice Solicitation and Team Voice Climate

**DOI:** 10.3389/fpsyg.2021.751446

**Published:** 2021-10-18

**Authors:** Rong Zhou, Wenjun Yin, Lin Sun

**Affiliations:** ^1^School of Management, Huazhong University of Science and Technology, Wuhan, China; ^2^School of Hotel Management, Guilin University of Tourism, Guilin, China

**Keywords:** leader narcissism, leader voice solicitation, team voice climate, team voice behavior, social information processing theory

## Abstract

Drawing on the narcissism literature and social information processing theory, we theorized and examined a serial mediation model linking leader narcissism with team voice behavior through leader voice solicitation and team voice climate. We tested our hypotheses using data collected from a time-lagged and multisource survey of 223 frontline employees in 60 teams at a large manufacturing organization. The results indicated that leader narcissism had a negatively indirect effect on team voice climate via leader voice solicitation. Team voice climate positively predicted team voice behavior, and the indirect effects of leader narcissism via leader voice solicitation and team voice climate on team voice behavior were significantly negative. In this paper, we discuss the theoretical implications of our findings for both the narcissism literature and the voice literature, along with their practical implications.

## Introduction

Voice behavior, defined as the discretionary expression of suggestions, ideas, opinions, or concerns about work-related issues in an attempt to constructively improve the current functioning of the unit or organization (Van Dyne and LePine, 1998; Morrison, 2011), has received increasing attention from scholars in the last several decades. In an ever more competitive and dynamic business environment, voice behavior is becoming increasingly important as a way for organizations to prompt effective decisions, swiftly identify potential problems, promote collective learning, and facilitate organizational innovation (Morrison, 2014; Bashshur and Oc, 2015). Scholars have also indicated that team voice behavior plays a vital role in organizations and demonstrates the real value of voice. They have claimed that compared with the isolated voice behavior of single employees, aggregated voice behavior exhibited by team members is more likely to be heard by leaders and thus to facilitate constructive change (Walumbwa et al., 2012; Frazier and Bowler, 2015). This study therefore focused on team voice behavior, defined as a team’s efforts to speak up to challenge the *status quo* and thereby promote constructive change.

Given the frequent interaction between team leaders and employees, leadership style and leader behavior can influence team members’ willingness to speak up about desired changes (Walumbwa et al., 2012; Frazier and Bowler, 2015; Liu H. et al., 2017; Ye et al., 2019). However, the role of leader narcissism in shaping team voice behavior is still unclear. Unlike conceptualized as a personality disorder in the clinical and psychiatric literature, narcissism is viewed as a personality trait in organizational research (Campbell et al., 2011) and such trait is quite common among leaders (Brunell et al., 2008; Nevicka et al., 2011a). Since Chatterjee and Hambrick (2007) first empirically investigated the influence of narcissistic CEOs on strategic dynamism and organization performance, research increasingly highlights the influence of leader narcissism on organization, team, and employees’ working attitudes and behaviors (Braun, 2017; Abatecola and Cristofaro, 2019; Cragun et al., 2020; Liu et al., 2021). In this study, we propose that it is necessary to delve into the potential impacts of leader narcissism on team voice behavior. To date, only one study has found that leader narcissism indirectly impedes team voice behavior through leader self-interested behavior when leaders’ unfairness perception is high (Liu H. et al., 2017). However, it should be noted that leader self-interested behavior is a broad negative behavior that may hinder various types of proactive behavior, not just voice behavior (Peng et al., 2019). Research has not thoroughly illustrated the specific voice-related process through which leader narcissism exerts an influence on team voice behavior.

To clearly focus on team voice issues in the workplace and explore the mechanisms underlying the impact of leader narcissism on team voice behavior, the current research constructed a serial mediation model that involved narcissistic leaders’ voice solicitation and team voice climate to explain how leader narcissism influences team voice behavior. Specifically, we proposed that the behavioral tendency for leader voice solicitation, namely the extent to which leaders proactively seek out suggestions for improvement and feedback from employees (Fast et al., 2014), is an immediate outcome of leader narcissism. Based on the narcissism literature, narcissistic leaders possess overconfidence in their ability to lead the team, are self-centered and disregard others’ thoughts, and strive to enhance and protect their grandiose, yet fragile, selves (Campbell and Foster, 2007). Narcissistic leaders with these characteristics tend to value their own decisions highly and avoid team members’ suggestions that they perceive to challenge their authority and competence. We thus expected leader narcissism to hinder leader voice solicitation. Leader voice solicitation can act as a salient cue that shapes employees’ collective beliefs about whether voice is encouraged in the workplace (i.e., team voice climate) (Lovelace et al., 2001; Morrison et al., 2011). Based on social information processing theory (Salancik and Pfeffer, 1978), narcissistic leaders’ low levels of voice solicitation convey that speaking up is not safe and not valued by team leaders. Accordingly, through social interaction team members shape the shared belief that voice is discouraged. This low level of team voice climate in turn hinders team voice behavior. We thus proposed that team voice climate is the distal mechanism through which leader narcissism and team voice behavior are linked. To test these hypotheses, we theoretically constructed and empirically examined a model of serial mechanisms to explain how leader narcissism exerts an impact on team voice behavior. This study collected data from a time-lagged and multisource survey of 223 frontline employees in 60 teams at a large manufacturing organization. We conducted path analysis in Mplus 8.3 to test our model. Results verified that leader narcissism hinders team voice climate via leader voice solicitation, and team voice climate is negatively related to team voice behavior. Furthermore, results indicated that leader narcissism impedes team voice behavior through the sequential mediation of leader voice solicitation and team voice climate.

Our research makes several important contributions. First, we contribute to the leader narcissism literature by testing a series of team-level mechanisms to understand how leader narcissism affects team voice behavior. Combining insights from the narcissism literature and social information processing theory, we provide an in-depth understanding of how leader narcissism impedes team voice behavior via voice-related processes involving leader behavior (i.e., leader voice solicitation) and team members’ shared perception of voice atmosphere (i.e., team voice climate). Second, our study extends research on how team voice climate develops in teams. Applying social information processing theory, we demonstrate that narcissistic leaders’ voice solicitation is a vital interpersonal antecedent of team voice climate, because it conveys signals about what attitudes and behaviors are encouraged. Our study thus answers the call for more empirical evidence of how leadership behaviors influence the emergence of a team voice climate (Morrison, 2011; Morrison et al., 2011; Frazier, 2013). Third, we offer additional evidence of the power of team voice climate to predict team voice behavior and enhance understanding of the indirect effects of negative leader behaviors on team voice behavior. We show that narcissistic leaders’ avoidance of voice solicitation from team members impedes the formation of team voice climate and in turn team voice behavior.

## Theory and Hypotheses

### Leader Narcissism and Leader Voice Solicitation

Narcissism originates from the ancient Greek mythology that a young man named Narcissus fell in love with his reflection in the pool and finally perished because of his over self-preoccupation (Campbell et al., 2011). In the clinical and psychiatric literature, narcissism is conceptualized as a personality disorder involving self-importance, self-focus, and grandiosity-based hostility toward others according to the *Diagnostic and Statistical Manual of Mental Disorders* (Grijalva and Harms, 2014). Organizational research has mainly focused on narcissism as a personality trait rather than a clinical disorder. Although the non-pathological narcissism has many characteristics that clinical narcissism has, individuals in the organizational research usually possess a subclinical level of narcissism (Campbell et al., 2011). In this research, we discuss narcissism as a personality trait that refers to a “complex of personality traits and processes that involve a grandiose yet fragile sense of self and entitlement as well as a preoccupation with success and demands for admiration” (Ames et al., 2006, p. 441). Based on Campbell and Foster (2007), narcissism involves three major characteristics: extreme positive sense of self, lack of empathy for others, and the use of self-regulatory strategies. Specifically, narcissists suppose that they are special, unique, and superior to others (Emmons, 1984; John and Robins, 1994). Narcissists’ self-evaluation is better than objective measures or others’ assessment of them, which reflects their overconfidence and inflated self-view (Judge et al., 2006). They also have a strong sense of entitlement and think of themselves as natural leaders (Ackerman et al., 2012). Narcissists’ high levels of grandiosity and self-importance in turn imply that they are excessively self-centered. Narcissists tend to show little empathy for others, disregard others’ needs, and prefer to strive for power and dominance than to forge and maintain intimate relationships (Emmons, 1987; Jordan et al., 2014). Moreover, people with narcissistic traits chronically engage in self-regulatory activities to construct and maintain their grandiose, yet fragile, selves. They continuously endeavor to enhance their self-view and protect themselves from being threatened through a set of behaviors such as dominating conversations, associating with high-status individuals, surpassing others via competition, denying others the recognition or appreciation they deserve, and taking credit from others (Morf and Rhodewalt, 2001; Campbell et al., 2011; Kjærvik and Bushman, 2021). These behaviors help narcissists reinforce their positive self-concept and obtain attention and admiration to achieve self-affirmation (Morf and Rhodewalt, 2001).

We expected that narcissists’ extreme positive sense of self and lack of empathy and self-regulatory processes would explain why narcissistic leaders are less likely to seek out suggestions and feedback from team members proactively (i.e., voice solicitation), based on the following evidence from the literature. First, narcissistic leaders tend to regard themselves as capable of managing teams effectively on their own; therefore, they believe that it is unnecessary to solicit input from team members (Judge et al., 2006; Kausel et al., 2015). As previously mentioned, due to their extreme positive sense of self, narcissistic leaders are excessively assertive and overconfident in their competence as leaders and perceive themselves as superior to their employees (Judge et al., 2006). Therefore, narcissistic leaders show a high level of confidence in their own judgment or decisions, even though their behavior often involves a high level of risk-taking with unpredictable performance (Campbell et al., 2004; Chatterjee and Hambrick, 2007). Second, narcissistic leaders’ traits of self-centeredness and having little empathy for employees inhibit their motivation to solicit voice from team members. Narcissistic leaders are inclined to focus on their own information and ideas and pay little attention to what team members think (Nevicka et al., 2011b). As voice solicitation from team members reduces narcissistic leaders’ opportunity to show off their self-perceived superiority (Han et al., 2020), narcissistic leaders are less motivated to ask for new ideas and suggestions from them. Third, avoiding voice solicitation can be regarded as a self-regulatory strategy that narcissistic leaders use to defend their grandiose yet fragile self. Although the voice behavior of team members may be future-oriented and constructive, they may also challenge the *status quo* (Van Dyne and LePine, 1998; Morrison, 2011). Narcissistic leaders with a fragile sense of self may fear that ideas, feedback, and suggestions solicited from team members will threaten their authority and competence as leaders (Burris, 2012; Fast et al., 2014; Zhang et al., 2021). Thus, narcissistic leaders tend to use defensive self-enhancement tactics such as avoiding voice solicitation from team members (Morrison and Milliken, 2000). This is consistent with the findings that leaders’ narcissism is negatively related to their change-oriented behaviors (Martin et al., 2016) and that narcissists are defensive about and resistant to feedback (Barry et al., 2006; Martinez et al., 2008). Taken together, we propose the following:

*Hypothesis 1: Leader narcissism is negatively related to leader voice solicitation.*

### Leader Voice Solicitation and Team Voice Climate

Organizational work climate reflects employees’ shared perceptions of the practices, policies, procedures, and behaviors that an organization expects, supports, and rewards (Kuenzi and Schminke, 2009; Schneider et al., 2013). Through frequent interaction and communication, employees attribute shared meaning to work events and form collective judgments of their work environment (Schneider and Reichers, 1983; Naumann and Bennett, 2000). Although organizational climate was originally defined as a global construct, scholars have highlighted multiple facet-specific climates, such as innovation climate (Scott and Bruce, 1994), justice climate (Colquitt et al., 2002), and service climate (Schneider et al., 1998). As our study focused on the specific voice context, we examined the development and effects of team-level voice climate in the workplace. Team voice climate represents team members’ shared perceptions of whether speaking up is encouraged or discouraged at work (Lovelace et al., 2001; Morrison et al., 2011). A strong voice climate within a team means that team members collectively hold the view that they can freely express doubts or concerns regarding the *status quo* to their leaders, and that their ideas will be taken seriously and valued. A weak voice climate, on the other hand, suggests that team members collectively believe that speaking up is undesirable and unacceptable (Morrison et al., 2011; Frazier and Fainshmidt, 2012).

Research has indicated that an organization’s work climate can be understood through social information processing theory. According to social information processing theory, employees construct their attitudes, cognition, and behaviors by processing social cues in the workplace (Salancik and Pfeffer, 1978). The social context provides cues about what work attitudes employees should hold and what behaviors are expected and accepted. Individuals focus on the most salient cues in their immediate social contexts and derive judgments from them about appropriate attitudes and actions (Salancik and Pfeffer, 1978). As immediate superiors interact frequently with employees and exert a vital impact on employees’ career development, these leaders are viewed as the most salient source of social information by employees (Priesemuth et al., 2014; Peng et al., 2019) and serve as “climate engineers” in the workplace (Zohar and Luria, 2005; Morrison et al., 2011). Thus, we draw on social information processing theory to explain the relationship between leader voice solicitation and team voice climate.

Leader voice solicitation refers to the extent to which team leaders proactively seek ideas, feedback, and suggestions from team members (Fast et al., 2014). We predicted that leader voice solicitation would help nurture team voice climate. When team leaders often solicit input from team members into how to improve team functioning, this provides team members with the positive signal that voice is welcomed and encouraged (Morrison et al., 2011; Carnevale et al., 2020). Through interpretation of leaders’ cues and communication with other members, team members form the shared perception that their leaders are open to and appreciate their input. As a result, they feel less at risk and uncertain in speaking up and believe that their ideas are more likely to be implemented (Tangirala and Ramanujam, 2012). However, when team leaders rarely solicit team members’ input, this signals that team leaders do not want team members to participate in team improvement and team decision making (Hunton et al., 1998). As voice behaviors can challenge the current practices and procedures, team members regard them as high risk (Morrison, 2011). Thus, team members regard speaking up as more risky and less effective when there is less voice solicitation from team leaders. Specifically, they anticipate negative consequences of voice such as punishment from leaders and the futility of speaking up (Morrison and Milliken, 2000). Taken together, we hypothesized as follows:

*Hypothesis 2: Leader voice solicitation is positively related to team voice climate.*

Combining Hypothesis 1 and Hypothesis 2, we expected leader narcissism to have a negative indirect effect on team voice climate via leader voice solicitation. As noted above, narcissistic leaders—characterized as possessing an extreme positive sense of self, lacking empathy, and constantly using self-regulatory strategies (Campbell and Foster, 2007)—have a behavioral tendency to avoid voice solicitation from team members. Such a low level of voice solicitation from narcissistic leaders may convey the signal that voice is not welcomed and not encouraged in teams (Salancik and Pfeffer, 1978; Schneider and Reichers, 1983). This further shapes a collective perception of weak team voice climate through communication and interaction among team members. Thus, we hypothesized as follows:

*Hypothesis 3: Leader voice solicitation mediates the effects of leader narcissism on team voice climate.*

### Effects on Team Voice Behavior

As voice behavior contains potential risks of challenging the organizational *status quo* and harming interpersonal relationships, a strong team voice climate can facilitate more team voice behavior. A strong team voice climate means that team members collectively believe that voice behavior is expected, supported, and rewarded in the workplace (Lovelace et al., 2001; Morrison, 2014). Consistent with social norms and expectations regarding voice, team members in such a climate are more likely to express their concerns, suggestions, and ideas to their leaders. A strong voice climate also indicates to team members that expressing their ideas is safe and effective, which enhances their willingness to speak up. However, a weak voice climate encourages team members to believe that speaking up is not expected or accepted (Lovelace et al., 2001; Morrison, 2014). Team members’ willingness to engage in voice behavior is thus inhibited because such behavior is perceived as risky, ineffective, and contrary to leaders’ expectations. Scholars have found that a strong team voice climate enhances individual-level voice behavior (Morrison et al., 2011) and team-level voice behavior (Frazier and Bowler, 2015). We thus hypothesized as follows.

*Hypothesis 4: Team voice climate is positively related to team voice behavior.*

Integrating Hypothesis 3 and Hypothesis 4, we expected leader narcissism to be associated with team voice behavior via leader voice solicitation and team voice climate. Combining the narcissism literature and social information processing theory (Salancik and Pfeffer, 1978; Campbell and Foster, 2007), a low level of voice solicitation from narcissistic leaders conveys a negative signal to team members that speaking up is risky and not encouraged, leading to a low level of voice climate in the team. This lower voice climate then guides team members to avoid engaging in voice behavior, which is perceived as inappropriate and undesired (Frazier and Bowler, 2015). Thus, we hypothesized as follows:

*Hypothesis 5: Leader narcissism has an indirect relationship with team voice behavior via leader voice solicitation and team voice climate.*

## Materials and Methods

### Sample and Procedures

To test our hypotheses, we carried out a two-wave multisource field survey in a large manufacturing company in Zhuzhou, China. With the help of the human resource director from this company, we collected data from leaders and frontline employees in equipment workshops at two time points. As frontline employees play an important role in finding problems during their daily work and voicing concerns and suggestions that are valuable to organizations, this was an appropriate context for our investigation. Participation was voluntary and all of the responses were kept confidential to decrease evaluation apprehension. At Time 1, team leaders gave reports on their narcissism traits. At the same time, employees reported their immediate leader’s voice solicitation and their demographic characteristics. A total of 238 employees and 64 team leaders completed the surveys. One month later (Time 2), the same leaders rated employees’ voice behaviors and employees assessed the voice climate in their teams. In total, we obtained valid matched data from 223 employees (valid response rate of 94%) nested in 60 teams. Of the 223 employees, 47.98% were female, the average age was 32.29 years (*SD* = 7.05) and the average organizational tenure was 6.76 years (*SD* = 5.29). The majority of the employees were well-educated: 77.13% of them had obtained a Bachelor’s degree or higher.

### Measures

All of the measures we used were originally developed in English. Following Brislin’s (1986) back-translation technique, we translated the English version of the scales into Chinese to ensure accuracy. Unless otherwise noted, responses to the items were given on a 7-point Likert scale ranging from 1 (*strongly disagree*) to 7 (*strongly agree*) for all of the scales.

#### Leader Narcissism

We measured leader narcissism using the nine-item narcissism scale developed by Jones and Paulhus (2014). Team leaders self-reported the extent to which they fitted the description of narcissism. A sample item is “I know that I am special because everyone keeps telling me so.” Cronbach’s alpha for this scale was 0.94.

#### Leader Voice Solicitation

Leader voice solicitation was measured using the four-item voice solicitation scale from Fast et al. (2014). The employees reported their perceptions of leader voice solicitation. A sample item is “My leader seeks out task-related knowledge from me.” Cronbach’s alpha for this scale was 0.89. To determine whether it was appropriate to aggregate the ratings for leader voice solicitation to the team level, we calculated intraclass correlation (ICC) values and the within-group interrater agreement (*R*_wg_). The ICC(1), ICC(2), and median *R*_wg_ were 0.60, 0.84, and 0.96, respectively—higher than the common criteria for the respective indexes (Bliese, 2000; LeBreton and Senter, 2008). We thus aggregated the employees’ ratings of leader voice solicitation to the team level.

#### Team Voice Climate

We measured team voice climate using Wei et al. (2015) three-item scale. The employees reported their perceptions of team voice climate. A sample item is “In my team, criticizing or providing information that challenges the feasibility of what is being done is encouraged.” Cronbach’s alpha for this scale was 0.73. As the ICC values and within-group interrater agreement (*R*_wg_) were acceptable, the average perception of team voice climate from employees in the same team represented the voice climate of that team (ICC[1] = 0.44, ICC[2] = 0.75, median *R*_wg_ = 0.89).

#### Team Voice Behavior

To assess team voice behavior, we first required the participating team leaders to assess their employees’ voice behavior using Van Dyne and LePine’s (1998) six-item, 5-point scale (1 = *strongly disagree* to 5 = *strongly agree*). A sample item is “This employee develops and makes recommendations concerning issues that affect this work team.” Cronbach’s alpha for this scale was 0.70. Next, we calculated the ICC values and the within-group interrater agreement (*R*_wg_). Because the *R*_wg_ index and ICC values were acceptable (ICC[1] = 0.41, ICC[2] = 0.72, median *R*_wg_ = 0.96), we proceeded to aggregate leader-rated employee voice behavior to the team level.

#### Control Variables

We controlled for team size and the average organizational tenure of each team, because these characteristics have been found to influence employee voice behavior (Hsiung, 2012; Lee et al., 2017) and team outcomes such as voice (Frazier and Bowler, 2015).

### Analysis Strategy

We conducted path analysis in Mplus 8.3 (Muthén and Muthén, 2012) to test our model of serial mediation. Specifically, leader narcissism was the independent variable, leader voice solicitation was the first-stage mediator, team voice climate was the second-stage mediator, and team voice behavior was the dependent variable. We examined our hypotheses and reported the results of testing the statistical significance of the indirect effects and the associated bootstrap analyses, based on 10,000 bootstrap samples with a 95% confidence interval (CI) (MacKinnon et al., 2002).

## Results

### Confirmatory Factor Analyses

We conducted a series of multilevel confirmatory factor analyses (MCFAs) with Mplus 8.3 to examine the distinctiveness of the core variables (leader narcissism, employee-rated leader voice solicitation, employee-rated team voice climate, and employee voice behavior). Before conducting the MCFAs, leader narcissism was packed into three parcels and employee voice behavior was packed into two parcels, as recommended by Little et al. (2013). The results indicated that the hypothesized four-factor model [χ^2^ = 112.402, *df* = 72, root mean square error of approximation (RMSEA) = 0.050, comparative fit index (CFI) = 0.944, Tucker–Lewis index (TLI) = 0.920, standardized root mean squared residual (SRMR)-within = 0.034, SRMR-between = 0.086] was a good fit to the data. This four-factor model fitted the data better than alternative three-factor models (e.g., combining employee-rated team voice climate and employee voice behavior, χ^2^ = 155.677, *df* = 77, RMSEA = 0.068, CFI = 0.890, TLI = 0.855, SRMR-within = 0.091, SRMR-between = 0.099), and two-factor models (e.g., combining employee-rated leader voice solicitation, employee-rated team voice climate, and employee voice behavior, χ^2^ = 235.100, *df* = 80, RMSEA = 0.093, CFI = 0.784, TLI = 0.725, SRMR-within = 0.126, SRMR-between = 0.136). Thus, the discriminant validity of the key constructs was confirmed.

### Descriptive Statistics and Correlations

The means, standard deviations, correlations, and reliabilities are displayed in Table 1. Leader narcissism was negatively related to leader voice solicitation (*r* = −0.49, *p* < 0.01), leader voice solicitation was positively related to team voice climate (*r* = 0.55, *p* < 0.01), and team voice climate was positively related to team voice behavior (*r* = 0.63, *p* < 0.01). These findings provided preliminary support for our hypotheses.

**TABLE 1:** Means, standard deviations, correlations, and reliabilities.

Variables	*Mean*	*SD*	1	2	3	4	5	6
1. Team size	3.72	0.49						
2. Average organizational tenure	6.74	3.22	0.03					
3. Leader narcissism	5.42	1.12	–0.06	–0.10	0.94			
4. Leader voice solicitation	2.94	1.01	0.19	0.23	−0.49**	0.89		
5. Team voice climate	3.68	1.03	0.26*	0.20	−0.41**	0.55**	0.73	
6. Team voice behavior	2.59	0.50	0.03	0.11	−0.45**	0.46**	0.63**	0.70

*p < 0.05; **p < 0.01. Coefficient alphas are shown in the diagonal.

### Hypotheses Tests

To verify our hypotheses, we performed a serial mediation path analytical model. As shown in Table 2, the team model path estimates supported our hypotheses. Hypothesis 1 posited that leader narcissism is negatively related to leader voice solicitation; the results in Table 2 supported this hypothesis (β = −0.47, *p* = 0.001). Hypothesis 2 posited that leader voice solicitation is positively associated with team voice climate, and it was also supported by the results (β = 0.41, *p* = 0.001). Hypothesis 3 suggested that leader voice solicitation mediates the effect of leader narcissism on team voice climate. Based on 10,000 bootstrap samples with a 95% CI, this hypothesis received empirical support (indirect effect = −0.19, 95% CI = [−0.370, −0.075]). Hypothesis 4 posited that team voice climate is positively related to team voice behavior. As shown in Table 2, this hypothesis was also supported (β = 0.54, *p* < 0.001). Hypothesis 5 posited that leader voice solicitation and team voice climate serially mediate the relationship between leader narcissism and team voice behavior. The results revealed that the indirect effect of leader narcissism on team voice behavior via leader voice solicitation (the first-stage mediator) and team voice climate (the second-stage mediator) was statistically significant (indirect effect = −0.10, 95% CI = [−0.233, −0.035]). We also found that the indirect effect of leader voice solicitation on team voice behavior via team voice climate was significant (indirect effect = 0.22, 95% CI = [0.078, 0.446]).

**TABLE 2:** Results of the path analysis.

Main effects	Leader voice solicitation	Team voice climate	Team voice behavior
Team size	0.16	0.17	–0.14
Average organizational tenure	0.18	0.08	–0.04
Leader narcissism	−0.47**	–0.19	–0.19
Leader voice solicitation		0.41**	0.10
Team voice climate			0.54***
*R* ^2^	0.30	0.36	0.47

**Indirect effects**	**Estimate**	**LLCI**	**ULCI**

Leader narcissism → Team voice climate (via leader voice solicitation)	−0.19*	–0.370	–0.075
Leader voice solicitation → Team voice behavior (via team voice climate)	0.22*	0.078	0.446
Leader narcissism → Team voice behavior (via leader voice solicitation and team voice climate)	−0.10*	–0.233	–0.035

*p < 0.05; **p < 0.01, ***p < 0.001. LLCI, lower level of the 95% confidence interval; ULCI, upper level of the 95% confidence interval.

## Discussion

Drawing on the narcissism literature and social information processing theory (Salancik and Pfeffer, 1978; Campbell and Foster, 2007), we explored the voice-related mechanisms that link leader narcissism and team voice behavior. Our results suggest that leader narcissism negatively impacts team members’ perceived leader voice solicitation, which in turn hinders the formation of a strong team voice climate. In addition, team voice climate positively influences team voice behavior. Team members are more likely to speak up and offer suggestions when they collectively perceive that they are encouraged to do so. Furthermore, we examined a sequential mediation model that revealed the effects of leader narcissism on team voice behavior via leader voice solicitation and team voice climate. The results confirmed our hypothesis that low levels of leader voice solicitation from narcissistic leaders serve as a significant cue signaling to teams that voice is not encouraged or acceptable. This weakens the team voice climate and ultimately inhibits team members’ voice behavior. Below, we discuss the theoretical implications of our findings.

Our research makes several notable theoretical contributions. First, it contributes to the narcissism literature by providing a nuanced understanding of how leader narcissism influences team voice behavior. Although some researchers have explored the impacts of leader narcissism on employee voice behavior (Carnevale J. B. et al., 2018; Carnevale J. et al., 2018; Huang et al., 2020), to the best of our knowledge only one empirical study has examined the relationship between leader narcissism and team voice behavior (Liu H. et al., 2017). Combining the perspectives of social exchange, social learning, and shifts in employees’ identity, Liu H. et al. (2017) found that when leaders’ unfairness perception was high, leader narcissism had a negative indirect influence on team voice behavior via leaders’ self-interested behavior. However, they did not specifically focus on the voice context to explore the voice-related processes influenced by leader narcissism. Leaders’ self-interested behavior is a general behavior derived from leader narcissism that can influence other proactive behaviors besides voice (Peng et al., 2019). To examine voice behavior specifically, our research identified two key mediating mechanisms that are closely related to voice (i.e., leader voice solicitation and team voice climate) underlying the relationship between leader narcissism and team voice behavior. Based on research on narcissists’ characteristics (e.g., Campbell and Foster, 2007), we found that narcissistic leaders tend to avoid soliciting input from team members to construct and maintain their grandiose but vulnerable egos. Based on social information processing theory (Salancik and Pfeffer, 1978), less voice solicitation from leaders conveys the signal to team members that voice is risky and not welcomed, weakening voice climate perception. This in turn makes team members unwilling to engage in voice behavior. We examined voice issues in the team context and verified the serial mediators that link leader narcissism and team voice behavior, providing a detailed understanding of the relationship between leader narcissism and team voice behavior.

Second, we add to voice climate research by exploring how team voice climate develops. Although scholars have emphasized the significance of team voice climate, they have rarely examined how such a climate develops. The literature has indicated that more empirical tests are needed to determine the role of leadership in the development of team voice climate (Morrison, 2011; Morrison et al., 2011; Frazier, 2013). Scholars have found that supervisor undermining hinders and leader humility facilitates the development of a team voice climate (Frazier and Bowler, 2015; Liu W. et al., 2017). We theorized and empirically demonstrated that leader narcissism exerts a detrimental indirect effect on team voice climate via leader voice solicitation. Based on social information processing theory, leader voice solicitation is a positive leader behavior that signals that voice behavior is welcomed and encouraged in teams, leading to a strong team voice climate. Leader narcissism indirectly hinders team voice behavior, because narcissistic leaders have a behavioral tendency to avoid soliciting suggestions from employees, sending out the negative signal that speaking up is discouraged. Our study answers the call to delve into additional leader factors associated with team voice climate (Morrison, 2011; Morrison et al., 2011; Frazier, 2013), advancing our understanding of how team voice climate emerges in the workplace. Our findings also support the argument in the climate literature that direct leaders have a powerful and proximal influence on team members’ climate perceptions (Zohar and Luria, 2005).

Third, our research contributes to the voice literature by providing extra support for the power of team voice climate to predict team voice behavior and extending the literature on how negative leadership influences team voice behavior. Consistent with the construct of team voice climate proposed by Morrison et al. (2011), our study indicates that team members do develop and share perceptions regarding voice behavior and that such shared beliefs guide team members’ subsequent voice behavior. We found that the stronger the shared perception that voice is encouraged, the less concerned team members are about the risks associated with voice and are more likely to express their concerns, suggestions, and ideas. This finding is in line with the results of Frazier and Bowler (2015). Furthermore, this study advances our understanding of the indirect effects of negative leader behaviors on team voice behavior. Our findings complement research on the effects of negative leader behaviors (i.e., supervisor undermining and leader self-interested behavior) on team members’ voice behavior (Frazier and Bowler, 2015; Liu H. et al., 2017) by highlighting the tendency of narcissistic leaders to avoid soliciting voice. Although leader voice solicitation promotes team voice behavior via team voice climate, leader narcissism may hamper this positive effect, as narcissistic leaders tend to avoid voice solicitation behavior.

## Conclusion and Implications

This study has some limitations that indicate directions for future research. First, although we collected data from two time points and different sources, we were still unable to make causal inferences from the results. Future researchers could conduct longitudinal or experimental design to replicate our findings. Second, our sample comprised frontline employees and their immediate leaders from a single manufacturing organization embedded in a homogenous cultural context, which limits the generalizability of our findings. We recommend that scholars further examine whether our hypotheses hold for other job types, industrial sectors, and cultural contexts. Third, although we examined two serial mediating mechanisms that link leader narcissism and team voice behavior, we did not delve into the boundary conditions for these mechanisms. Future research could pay more attention to the contextual conditions that attenuate or amplify the behavioral expressions of narcissistic leaders. As the results showed, narcissistic leadership had a negative indirect effect on team voice climate through reduced leader voice solicitation. However, there are some situations in which narcissistic leaders may be willing to proactively seek suggestions from employees. For example, resource conflict with another team can increase internal cooperation and resource contribution (Van Bunderen et al., 2018), and team leaders tend to prioritize collective goals when facing intense competition with other teams (Maner and Mead, 2010). Thus, conflict or competition with another team may mitigate the relationship between leader narcissism and leader voice solicitation, and in turn help promote the development of team voice climate and more team voice behavior. Fourth, although we explored the potentially detrimental effects of leader narcissism on team voice behavior in general, leader narcissism may differ in its negative influence on different types of voice as promotive and prohibitive voice. Prior research has indicated that promotive voice emphasizes employees’ expression of improving the *status quo* while prohibitive voice emphasizes employees’ expression of criticizing the *status quo* (Liang et al., 2012). As team prohibitive voice reflects leaders’ incompetence to a greater extent than team promotive voice and thus heavily threatens narcissistic leaders’ grandiose yet fragile self, the negative indirect effect of leader narcissism on team prohibitive voice may be stronger. Similarly, inter-team competition may temper or even reverse such detrimental influence as narcissistic leaders need both promotive and prohibitive suggestions to help win the competition between teams. Finally, as we focused on team voice issues in the workplace, we examined leader voice solicitation as the antecedent of voice climate and team voice behavior as the outcome of voice climate. It would be useful to identify other antecedents and outcomes associated with voice climate. As research has indicated, voice climate may derive from team members’ exchange relationships and influence team performance, team innovation, and employee service performance as well (Frazier and Fainshmidt, 2012; Frazier and Bowler, 2015; Duan et al., 2019). Future research could combine the leader factors and team member factors to explore the development of voice climate. It could also consider other team-level outcomes of voice climate, such as team cohesion, alongside other individual-level outcomes, such as job satisfaction.

The findings of this research indicate that leader narcissism can indirectly hinder the development of team voice climate and team members’ voice behavior, and that leader voice solicitation can indirectly facilitate team members’ engagement in voice behavior. These results have important practical implications for both managers and organizations. For managers, because narcissism is strongly positively related to leader emergence (Brunell et al., 2008; Nevicka et al., 2011a), it may be difficult to prevent narcissistic individuals from emerging as leaders. Our results suggest that managers’ behaviors are socially perceived by employees and can guide employees’ behaviors; therefore, narcissistic managers should constantly monitor and reflect on their behaviors (Maccoby, 2004), and pay attention to how team members perceive their behaviors. This self-initiated reflection would help narcissistic managers change their typical dysfunctional behavioral tendencies into productive leadership behaviors. For organizations, our findings suggest that managers should receive more training in self-development and self-monitoring. Such training could encourage and guide narcissistic managers to reflect on their daily behaviors (Frazier and Bowler, 2015). In addition, as leader voice solicitation can help shape a high level of voice climate perception, which in turn promotes team members’ voice behavior, organizations should highlight the importance of proactively soliciting employees’ ideas and suggestions for organizational development during leadership training (Carnevale J. B. et al., 2018). This would support the emergence of a voice climate and enhance employees’ willingness to speak up.

## Data Availability Statement

The raw data supporting the conclusions of this article will be made available by the authors, without undue reservation.

## Ethics Statement

Ethical review and approval was not required for the study on human participants in accordance with the local legislation and institutional requirements. The patients/participants provided their written informed consent to participate in this study.

## Author Contributions

RZ wrote the manuscript. WY helped with the manuscript revision. LS collected the data. All authors contributed to the article and approved the submitted version.

## Conflict of Interest

The authors declare that the research was conducted in the absence of any commercial or financial relationships that could be construed as a potential conflict of interest.

## Publisher’s Note

All claims expressed in this article are solely those of the authors and do not necessarily represent those of their affiliated organizations, or those of the publisher, the editors and the reviewers. Any product that may be evaluated in this article, or claim that may be made by its manufacturer, is not guaranteed or endorsed by the publisher.
